# Usability of the Level of the S100B Protein, the Gosling Pulsatility Index, and the Jugular Venous Oxygen Saturation for the Prediction of Mortality and Morbidity in Patients with Severe Traumatic Brain Injury

**DOI:** 10.1155/2021/2398488

**Published:** 2021-10-25

**Authors:** Ryszard Tomasiuk, Sebastian Dzierzęcki, Artur Zaczyński, Mirosław Ząbek

**Affiliations:** ^1^Kazimierz Pulaski University of Technology and Humanities Radom, Faculty of Medical Sciences and Health Sciences, Radom, Poland; ^2^Department of Neurosurgery, Postgraduate Medical Centre, Warsaw, Poland; ^3^Gamma Knife Centre, Brodno Masovian Hospital, Warsaw, Poland; ^4^Clinical Department of Neurosurgery, Central Clinical Hospital of the Ministry of the Interior and Administration, Warsaw, Poland

## Abstract

The high frequency of traumatic brain injury imposes severe economic stress on health and insurance services. The objective of this study was to analyze the association between the serum S100B protein, the Gosling pulsatility index (PI), and the level of oxygen saturation at the tip of the internal jugular vein (SjVO2%) in patients diagnosed with severe TBI. The severity of TBI was assessed by a GCS score ≤ 8 stratified by Glasgow outcome scale (GOS) measured on the day of discharge from the hospital. Two groups were included: GOS < 4 (unfavorable group (UG)) and GOS ≥ 4 (favorable group (UG)). S100B levels were higher in the UG than in the FG. PI levels in the UG were also substantially higher than in the FG. There were similar levels of SjVO2 in the two groups. This study confirmed that serum S100B levels were higher in patients with unfavorable outcomes than in those with favorable outcomes. Moreover, a clear demarcation in PI between unfavorable and FGs was observed. This report shows that mortality and morbidity rates in patients with traumatic brain injury can be assessed within the first 4 days of hospitalization using the S100B protein, PI values, and SjVO2.

## 1. Introduction

The leading global causes of death are traumatic brain injury (TBI), cardiovascular disease, and cancer [1].

TBI includes noncongenital tissue damage rendered by a sudden impact manifested by endocrine dysfunction, electrolyte imbalance, respiratory manifestations, and neurological, neuropsychological, and psychiatric dysfunction [2]. TBI occurs most frequently in two age groups: between 15 and 24 and older than 75 years of age [3]. Although TBI is prevalent internationally at 1.3 and 2 per 100,000 in North America and Europe, respectively, in Poland, its frequency oscillates around 0.07 per 100,000. This high frequency of TBI imposes severe economic stress on health and insurance services due to costly and complicated treatment and rehabilitation processes [4–6].

Methods used for the diagnosis and treatment of TBI include the analysis of biochemical markers [7, 8], transcranial Doppler testing (TCD) [9], measurement of cerebral perfusion pressure (CPP) [10], the Glasgow Coma Scale (GCS) [11], and Marshall score [12].

To date, a variety of markers have been used to assess the severity of TBI, including lactate dehydrogenase [13], myelin alkaline protein [14], neuron-specific enolase [15], and creatine kinase [16]. However, none have been shown to be suitable for clinical practice. Recently, the S100B protein that is present in detectable concentrations in blood, serum, and cerebrospinal fluid (CSF) has attracted the interest of researchers. Its changes in serum level reflect the degree of posttraumatic brain damage, making it an adequate means for assessing the level of TBI [17–19]. S100B is crucial in intracellular processes of cell growth and metabolism [20], and its secretion induces autocrine and paracrine effects on glial cells, microglial cells, and neurons [21]. At micromolar concentrations, S100B induces apoptosis [18].

Other brain injury diagnosis methods include GCS, CPP, and TCD. Thus, GCS allows for an objective assessment of consciousness impairment in acute medical and trauma patients. TCD is a noninvasive technique that allows real-time monitoring of CPP, intracranial pressure (ICP), and cerebral blood flow (CBF). CPP is measured using the pulsatility index (PI) values of the middle cerebral artery (MCA). The CBF level is assessed using the mean blood flow velocity (MFV) [22].

Since the effectiveness of TBI treatment impacts patient outcome and reflects the quality of TBI diagnosis, there is ongoing research to find a “golden” TBI severity predictor or a method for quick and error-free diagnosis.

Considering the complexity of the problem and the continuing clinical demand, we conducted a study on the associations between serum S100B, PI, and the level of oxygen saturation at the tip of the internal jugular vein (SjvO2) in patients diagnosed with severe TBI (defined by a GCS score ≤ 8).

## 2. Methods

### 2.1. Study Subjects

The Bioethics Committee of the Medical Center of Postgraduate Education in Warsaw approved the experimental protocols. All subjects or their guardians and if subjects were under 18, a parent and/or legal guardian signed an informed consent.

After admission to the Department of Neurosurgery and Trauma of the Nervous System, the patient's health was assessed using the GSC [23, 24]. Each patient was also subjected to the standard diagnostic and therapeutic protocol in accordance with the European Consortium of Brain Injury Guidelines ([25] Patients with poor ventilation underwent a gasometric examination to optimize pCO_2_ (range 30–40 mmHg) and maintain hematocrit (Ht) and hemoglobin (Hb) levels at 30–40% and 12–14 g/dL, respectively.

**Figure 1:**
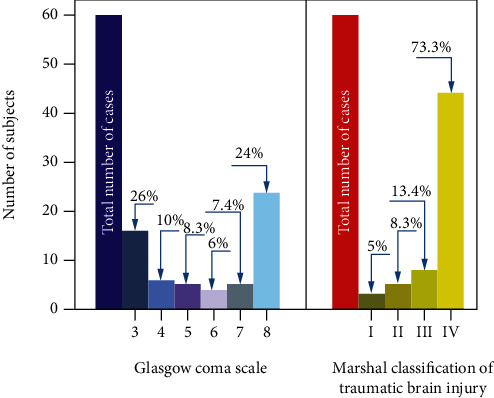
Graphical representation of patients admitted to the Medical Centre of Postgraduate Education. (a) Distribution of subjects GCS ≤ 8. (b) Marshall classification of traumatic brain injury (MCTC) distribution. Numerals above the bars correspond to the number of specific cases (percentage of cases).

Only patients with a GCS score ≤ 8 were included in the study. The study group consisted of 60 patients (48 men and 12 women); the GCS and Marshall scale distributions are shown in Figure 1.

Venous blood samples (5 mL) were collected at admission and at 24-hour intervals for another 96 h. Pretreated blood samples (clotted and centrifuged for 10 min at 1,000 RPM) were stored at -22°C. Protein concentration measurements were performed using a Liason Sangtec 100 kit with a broad diagnostic spectrum of 0.02–30 *μ*g/L and a sensitivity threshold of 0.02 *μ*g/L.

PI [26] was derived using a transcranial Doppler examination performed on a Mediasonic Transpect CDS Doppler in power motion mode TCD (PMD/TCD) at 24-hour intervals for 96 h after admission to the Neurosurgery Clinic. Initially, the arteries of the brain base accessible through the temporal window were scanned. Further analysis was completed on the middle cerebral arteries on the dominant lesion's side or the right side if the lesion's extent was symmetrical.

SjvO2 was determined in blood samples drawn from a catheter positioned in the jugular bulb using an oximeter (IL-284 CO-Oximeter; Instrumentation Laboratory, Lexington, MA).

The self-assessed S100B and PI reference ranges were determined using a group of 40 healthy volunteers: 25 men and 15 women with an average age of 47.0 ± 14.77 years (age range of 21 to 80 years).

### 2.2. Statistical Analysis

A sample normality was evaluated using the Shapiro-Wilk test [27]. Data are expressed as mean ± standard deviation, with minimum and maximum values. Differences in the means of the study groups, i.e., “favorable” and “unfavorable,” at specific times, were tested using a bootstrapped test for differences in means of 10,000 repeats with replacement [28]. Changes in S100B, PI, and SjvO2 levels stratified by the study group were computed using the one-way Aligned Rank Transform for Nonparametric Factorial ANOVAs (ART) technique [29]. Because of the shortcomings of current statistical methods in handling advanced nonparametric statistics, we discuss only the outcome of one-way nonparametric factorial ANOVA. Patient mortality was taken into account by censoring the number of subjects in a group. The rate of change of a specific parameter was evaluated using the slope of a linear regression model. Values of *P* < 0.01 were considered statistically significant. We conducted all analyses in the R programming language .

## 3. Results

This study was performed on two groups of patients stratified by GOS [30] and assessed on the day of discharge from the hospital. The UG consisted of patients with GOS < 4. FG group encompassed patients with GOS ≥ 4. The stratification scheme led to the *post hoc* assignment of 51 and 9 patients to the UG and FG, respectively. The average age in the UG was 48 years (19-73) and 47 years (14–75) in the FG.

**Figure 2:**
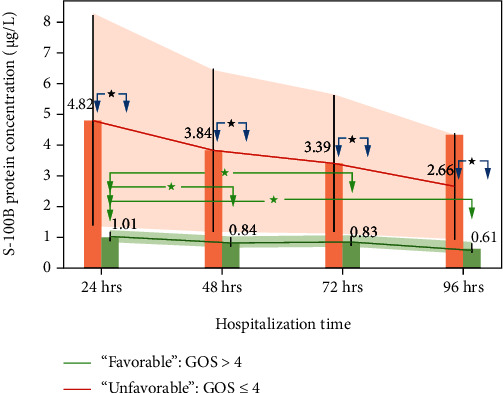
Changes in S100B protein level stratified by GOS as a function of hospitalization time. Whiskers represent the standard error; above the bar are the mean values at the specific time. Red arrows correspond to statistically significant changes between measurements at specific times in the “unfavorable” group. Green arrows correspond to statistically significant changes between measurements at specific times in the “favorable” group. Shaded regions correspond to 95% confidence intervals for the “unfavorable” group, red, and “favorable” group, green. ^∗^ refers to *P* < 0.01.

The standard reference range for serum S100B protein concentration was 0.05–0.23 *μ*g/L. The changes in the levels of the S100 protein of the studied groups (measured at 24, 48, 72, and 96 h after hospital admission) are compiled in Table 1 and Figure 2.

Figure 2 shows that S100B levels in UG were generally higher across all measurements than those in FG. In UG, a decreasing rate of *V*_S100B_U_ = 0.03 *μ*g/L/h was observed. However, there were no statistically significant changes between consecutive measurements. In FG, statistically significant changes in S100B levels were observed between 24 and 48, 24 and 72, and 24 and 96 h, and the rate of decrease in the S100B level was *V*_S100B_F_ = −5.56∗10^−3^ *μ*g/L/h.

**Figure 3:**
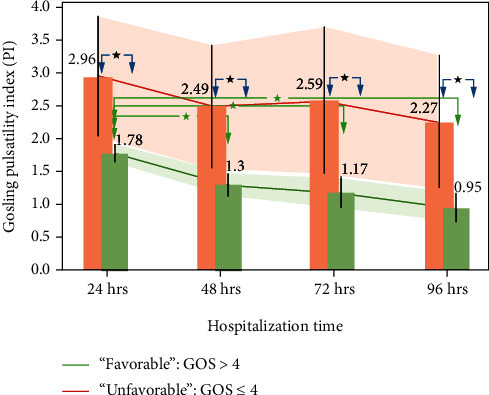
Changes in the Gosling pulsatility index (PI) stratified by GOS as a function of hospitalization time. Whiskers represent the standard error; above the bar are the mean values at the specific time. Red arrows correspond to statistically significant changes between measurements at specific times in the “unfavorable” group. Green arrows correspond to statistically significant changes between measurements at specific times in the “favorable” group. Shaded regions correspond to 95% confidence intervals for the “unfavorable” group, red, and “favorable” group, green. ^∗^ refers to *P* < 0.01.

PI changes, which reflect cerebral flow, are compiled in Table 2 and Figure 3. We observed that PI levels in UG patients were substantially higher than in FG patients. Moreover, FG was defined by a decrease in PI level of *V*_PI_U_ = −9.58∗10^−3^/h and the lack of statistically significant differences between consecutive measurements. In FG, statistically significant changes occurred between 24 and 48 and 24 and 72 h of hospitalization, and the rate of decrease in PI was *V*_PL_F_ = −0.01 cm/s/h. The relative ratio of the unfavorable to favorable group PI decrease rate was close to one: *V*_PI_U_/*V*_PI_F_ = 9.58∗10^−3^/10^−2^ = 0.96.

**Figure 4:**
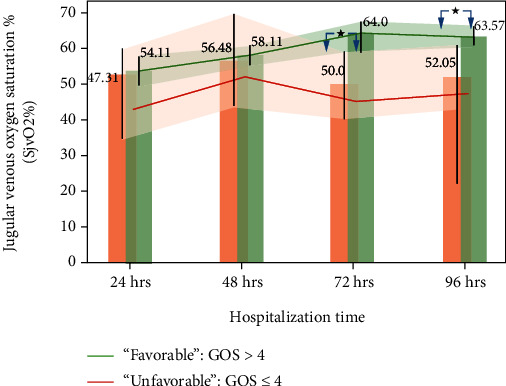
Changes in SjvO2% stratified by GOS as a function of hospitalization time. Whiskers represent the standard error; above the bar are the mean values at the specific time. Red arrows correspond to statistically significant changes between measurements at specific times in “unfavorable” group. Green arrows correspond to statistically significant changes between measurements at specific times in “favourable” group. Shaded regions correspond to 95% confidence intervals for “unfavorable” group, red, and “favorable” group, green. ^∗^ refers to *P* < 0.01.

The fluctuations in SjvO2% are shown in Table 3 and Figure 4. The analysis of Figure 4 showed similar levels of SjVO2 in both groups. However, at 72 and 96 h, the differences in SjvO2% between UG and FG were statistically significant. The rate of decrease in SjvO2% in UG was *V*_SjvO2_U_ = −0.06%/h and in FG was *V*_SjvO2_F_ = −0.13%/h. The relative decrease rate of SjvO2 between the respective groups was 0.46.

## 4. Discussion

TBI diagnosis and treatment methods are still insufficient and result in a mortality rate of 30–60% [31–40]. In recent decades, the development of diagnostic techniques that exploit the physical and biochemical functions of the human body has led to the use of biochemical markers [7, 8], TCD [9], and CPP measurement [10] as the primary means of clinical diagnosis in patients with TBI.

To date, several analyses have been reported on the usability of S100B protein levels in assessing the severity of TBI [41–44]. However, none performed a combined analysis of the levels of S100B protein, Gosling's pulsatility index, and jugular venous oxygen saturation for the prediction of mortality and morbidity among patients with TBI. To our knowledge, this study is a unique approach that allows for a more accurate assessment of the severity of TBI and prognosis related to the modality of treatment.

One study showed that S100B, *V*_mean_ levels, and SjvO2% are verifiable TBI severity markers [35]. This study verified the usability of an early analysis, 1 to 4 days after hospital admission, of changes in S100B, *V*_mean_, and SjvO2% levels for prognostic properties in clinical diagnosis. To overcome the limitation of this study caused by a small sample size, differences between studied groups were assessed by bootstrap analysis [45, 46]. Thus, through the introduction of 10,000 repeats in bootstrap analysis, we were able to achieve a good representation of the population according to the literature.

The study was performed on two samples stratified by GOS < 4 (unfavorable group (UG)) and GOS ≥ 4 (favorable group (FG)). The 26.6% mortality reported in this study was greater than the 13% reported by Gerber et al. [47]. Standard S100B levels oscillated between 0.05 and 0.23 *μ*g/L and were considerably different from those previously reported as pathological [48].

Our results generally agree with the report by Raabe and Seifert [48], showing that serum levels of S100B are higher in patients with unfavorable outcomes than in those with favorable outcomes. However, we did not confirm an early stage increase in S100B levels in FG. Furthermore, this study showed that the increased levels of S100B in FG patients did not return to normal during the first four days of hospitalization and were on average 0.61 mg/L on day 4. The decreasing rate of S100B levels in UG was equal to 5.655 *μ*g/L/h, indicating that the nonpathological range might have been reached 153 h after hospital admission and 30 h longer than the value observed for FG. This observation indicates the presence of irreversible pathological changes that occur in brain tissue during the additional 30 h required to reach the reference range.

TCD allows the analysis of abnormalities in cerebral circulation in patients with TBI [22, 49–52] manifested by a decrease in *V*_mean_ and an increase in PI [53, 54]. *V*_mean_ reference levels determined in this study [55] agreed with others [56–58] and defined the abnormal level of PI as ≥1.15. UG was defined by PI levels greater than 1.94 at the end of the first day of hospitalization. There was a clear demarcation in PI between UG and FG. This observation is in agreement with other reports [59–61].

We also confirmed high mortality in patients with elevated PI. Moreno et al. [62] reported 83% mortality for patients with an initial PI > 1.5 and 100% for those with an initial PI > 2.6. Though mortality in patients with PI > 1.57 was only 27%, it is still an important factor in TBI treatment. Thus, the complexity of the problem did not allow us to speculate on the causes of the observed mortality differences.

Analysis of the rate of decrease in PI indicated that the time required to reach the normal threshold was 74 h longer in UG than in FG. Therefore, PI levels in UG would have required 7 days to reach the “safe zone,” while PI levels in FG dropped significantly after 24 h of hospitalization, resulting in mean PI values below the threshold equal to 1.5.

Analysis of jugular venous oximetry (SjvO2%), a measurement of the balance between metabolic demand, cerebral oxygen supply, and cerebral metabolic demand [63, 64] in patients with TBI, allows assessment of the severity of trauma, including cerebral hypoperfusion or ischemia [65–67]. Although a recent study showed normal SjvO2% between 44.7 and 69.5% [63], the generally accepted range is between 55 and 75% [68]. Thus, for discussion purposes, we employ the latter.

This study showed SjvO2% near and above 55% for nearly all patients in FG. Furthermore, there was an increase in SjvO2% at a rate of 0.13%/h from 24 to 96 h. UG was delineated by an SjvO2% below 55% and a minuscule rate of change of 0.06%/h, reflecting, most probably, irreversible ischemic desaturation. Furthermore, the mortality rate rendered by unfavorable SjvO2% was greater than previously reported [66]: 27% versus 17%.

An amalgam of this and a previous study [69] indicates that SjvO2% may be used to optimize TBI treatment. However, when evaluating the positive influence of an increase in SjvO2%, caution must be exercised. Yokota et al. [70] showed that the mortality rate is a function of a dramatic increase in the SjVO2% level, reaching 100% for cases defined by an SjvO2% greater than 90% above the standard threshold. Unfortunately, we were unable to assess the minimum rate of increase in SjvO2%, resulting in a detrimental result.

An analysis of correlations between the parameters studied indicated that FG was defined by a statistically significant decrease in S100B protein levels correlated with a statistically significant decrease in PI and a small increase in SjVO2%. Furthermore, all parameters were within nonpathological ranges within 6 days of the patient's admission. The changes observed in the UG were in the same direction but of greater magnitude than those in the FG. The levels of the respective parameters were significantly higher in UG than in FG and did not reach “normality” for at least 7 days after the patient's admission.

## 5. Conclusions

In conclusion, mortality and morbidity rates in patients with TBI can be assessed within the first 4 days after hospitalization using S100B protein and PI levels and SjVO2%. Extension of the presented research should lead to the development of improved personalized TBI treatment, greatly increasing the chance of patient survival.

## Figures and Tables

**Table 1:** Changes in S-100b protein level stratified by GOS as a function of hospitalization time.”UG” – “unfavorable” group, “FG” – “favorable” group.

outcome	measurement x 24 hrs	mean (mg/L)	STD (standard deviation)	min (mg/L)	max (mg/L)	N (number of subjects)
UG	1	4.82	4.45	0.76	19.8	51
	2	3.84	4.21	0.47	16.8	48
	3	3.39	4.04	0.38	20.83	40
	4	2.66	3.05	0.136	16.7	37

FG	1	1.01	0.29	0.71	1.6	9
	2	0.84	0.21	0.62	1.3	9
	3	0.83	0.35	0.51	1.51	9
	4	0.61	0.24	0.39	1.1	9

**Table 2:** Changes in PI stratified by GOS as a function of hospitalization time. UG” – “unfavorable” group, “FG” – “favorable” group.

outcome	measurement x 24 hrs	mean (cm/s)	STD (standard deviation)	min (cm/s)	max (cm/s)	N (number of subjects)
UG	1	2.96	1.10	1.2	7.1	51
	2	2.49	1.18	1.06	6.11	48
	3	2.59	1.39	0.91	6.78	40
	4	2.27	1.40	0.89	6.8	37

FG	1	1.78	0.16	1.5	2.11	9
	2	1.30	0.17	1.1	1.6	9
	3	1.17	0.26	0.91	1.8	9
	4	0.98	0.31	0.63	1.6	9

**Table 3:** Changes in SjvO2 stratified by GOS as a function of hospitalization time. UG” – “unfavorable” group, “FG” – “favorable” group.

outcome	measurement x 24 hrs	mean (%)	STD (standard deviation)	min (cm/s)	max (cm/s)	N (number of subjects)
UF	1	47.31	20.51	21	98	51
	2	56.48	17.85	20	95	48
	3	50.00	18.33	22	97	40
	4	52.05	18.21	17	99	37

UG	1	54.11	13.23	37	69	9
	2	58.11	6.68	49	72	9
	3	64.00	8.89	51	81	9
	4	63.57	8.06	50	73	7

## Data Availability

Data are provided on request.
